# Inequalities in out-of-pocket payments for health care services among elderly Germans – results of a population-based cross-sectional study

**DOI:** 10.1186/1475-9276-13-3

**Published:** 2014-01-08

**Authors:** Jens-Oliver Bock, Herbert Matschinger, Hermann Brenner, Beate Wild, Walter E Haefeli, Renate Quinzler, Kai-Uwe Saum, Dirk Heider, Hans-Helmut König

**Affiliations:** 1 Department of Health Economics and Health Services Research, Hamburg Center for Health Economics, University Medical Center Hamburg-Eppendorf, Martinistrasse 52, Hamburg 20246, Germany; 2 Institute for Social Medicine, Occupational Health and Public Health, University of Leipzig, Philipp-Rosenthal-Straβe 55, Leipzig 04103, Germany; 3 Division of Clinical Epidemiology and Aging Research, German Cancer Research Center, Im Neuenheimer Feld 581, Heidelberg 69120, Germany; 4 Department of General Internal Medicine and Psychosomatics, Heidelberg University Hospital, Thibautstrasse 2, Heidelberg 69115, Germany; 5 Department of Clinical Pharmacology and Pharmacoepidemiology, University of Heidelberg, Im Neuenheimer Feld 410, Heidelberg 69120, Germany

**Keywords:** Out-of-pocket payments, Inequalities, Germany, Elderly

## Abstract

**Introduction:**

In order to limit rising publicly-financed health expenditure, out-of-pocket payments for health care services (OOPP) have been raised in many industrialized countries. However, higher health-related OOPP may burden social subgroups unequally. In Germany, inequalities in OOPP have rarely been analyzed. The aim of this study was to examine OOPP of the German elderly population in the different sectors of the health care system. Socio-economic and morbidity-related determinants of inequalities in OOPP were analyzed.

**Methods:**

This cross-sectional analysis used data of N = 3,124 subjects aged 57 to 84 years from a population-based prospective cohort study (ESTHER study) collected in the Saarland, Germany, from 2008 to 2010. Subjects passed a geriatric assessment, including a questionnaire for health care utilization and OOPP covering a period of three months in the following sectors: inpatient care, outpatient physician and non-physician services, medical supplies, pharmaceuticals, dental prostheses and nursing care. Determinants of OOPP were analyzed by a two-part model. The financial burden of OOPP for certain social subgroups (measured by the OOPP-income-ratio) was investigated by a generalized linear model for the binomial family.

**Results:**

Mean OOPP during three months amounted to €119, with 34% for medical supplies, 22% for dental prostheses, 21% for pharmaceuticals, 17% for outpatient physician and non-physician services, 5% for inpatient care and 1% for nursing care. The two-part model showed a significant positive association between income (square root equivalence scale) and total OOPP. Increasing morbidity was associated with significantly higher total OOPP, and in particular with higher OOPP for pharmaceuticals. Total OOPP amounted to about 3% of disposable income. The generalized linear model for the binomial family showed a significantly lower financial burden for the wealthiest quintile as compared to the poorest one.

**Conclusions:**

This is the first study providing evidence of inequalities in OOPP in the German elderly population. Socio-economic and morbidity-related inequalities in OOPP and the resulting financial burden could be identified. The results of this study may contribute to the discussion about the mechanisms causing the observed inequalities and can thus help decision makers to consider them when adapting future regulations on OOPP.

## Introduction

### Background

Health expenditure has increased in virtually all countries of the Organization for Economic Co-operation and Development (OECD) over the last years [1]. In order to limit rising publicly-financed health expenditure, out-of-pocket payments for health care services (OOPP) were introduced or raised in many OECD countries [2]. There are two main goals of OOPP. Firstly, they are intended to reduce inefficiencies that could result from the phenomenon that is referred to as ‘moral hazard’. The idea behind this is that patients might tend to utilize health care services too frequently when they are free of charge. By being forced to contribute to health care costs directly, patients may reduce their health care utilization. Secondly, OOPP can serve as a crude means of financing the health care system without increasing taxes or social security contributions. This aspect becomes more and more important since many OECD countries are in a general tight budgetary situation.

However, the implementation of patients’ direct contribution to health care costs entails the danger of burdening different social sub-groups unequally. Therefore, the question of especially income-, gender- and education-related inequalities in OOPP has often been examined, mostly focusing on the elderly due to their higher health-related resource utilization [3].

Nonetheless, corresponding evidence from Germany, the third largest OECD economy [4], is missing. Germany has one of the most expensive health care systems both in absolute numbers and compared to its gross domestic product. Additionally, Germany has one of the oldest populations in the world with a large, still growing proportion of elderly people [5]. Within the German health care system, there is a comprehensive system of OOPP.

### Regulation of OOPP in Germany

Approximately 88% of the German population is insured by a statutory health insurance (SHI). Compulsory beneficiaries of the German SHI are especially employees under a certain income threshold. Self-employed, and employees above the threshold can choose between SHI and private health insurance (PHI). Civil servants, pensioners and their families profit moreover from governmental schemes (GS) that cover a percentage of the health-related financial risk so that a PHI only has to cover the residual risk. Regardless of the type of health insurance, all beneficiaries have access to comprehensive health care services, including out- and inpatient services and pharmaceuticals.

As the vast majority of the German population is in a SHI, following explanations will focus on the regulations of OOPP in the SHI from 2008 to 2010, the time of data collection of this study. OOPP existed in the German SHI for all health-care services from 2008 to 2012 without regulatory changes [6]. Table 1 gives an overview about the regulation of OOPP in the SHI in the relevant sectors. Additionally to these regulations, a beneficiary had to pay maximally OOPP up to the amount of 2% of his or her gross income. This rule was designed to limit the potential financial burden due to OOPP. If a beneficiary was chronically ill, which was proven by having the same diagnosis over several quarters, he or she had to pay up to only 1% of the gross income. The 1%- or 2%- threshold only became effective upon request at one’s SHI. Yet, the limitations did not apply for dental prostheses and goods or services that were not reimbursed by the SHI, e.g. non-prescription drugs and glasses.

**Table 1:** Overview: OOPP within German statutory health insurance (2008–2012)

**Resource**	**Regulation of OOPP**
Inpatient care	10 Euro per day for not more than 28 days in hospitals and follow-up-rehabilitations.
Outpatient services	Physicians: 10 Euro per quarter for the first contact with a general practitioner or specialist; Dentists: 10 Euro per quarter for the first contact; Non-Physicians: 10 Euro per prescription and 10% of costs.
Medical supplies	Permanent use: 10% of costs but not more than 10 Euro per month; single use: 10% of costs not less than 5 Euro and not more than 10 Euro; glasses are not reimbursed.
Pharmaceuticals	10% of costs but not less than 5 Euro and not more than 10 Euro per drug; non-prescription drugs are not reimbursed.
Dental prostheses	SHI reimburses 50% of costs, in case of regularly check-ups in the last 5 (10) years 70% (80%).
Nursing care	10 Euro per prescription plus 10 Euro per day for the first 28 days.

### Aim

The aim of this study was to determine the amount of OOPP elderly people pay in the different sectors of the German health care system. The elderly were considered due to their high and continually growing importance for the German health care system. Furthermore, socio-economic and morbidity-related determinants of inequalities in OOPP were analyzed. In addition, the financial burden on various social sub-groups was examined.

## Methods

### Sample

Data were collected within the eight-year follow-up wave of the ESTHER study, a large population-based prospective cohort study consisting of 9,949 people aged between 50 and 74 who were recruited from 2000 to 2002 in the Saarland, Germany. From baseline to this eight-year follow-up, 499 subjects had died, 505 individuals were no longer able to participate due to poor health, and 680 had declined further participation. From the remaining 8,265 participants, 6,063 (73.4% response rate) sent back a detailed questionnaire of the eight-year follow-up. Further additional information from a questionnaire provided by their general practitioners was available for 5,056 individuals, and 3,124 participants consented to an additional three hour geriatric assessment performed by study physicians at their homes. The geriatric assessments were conducted between July 2008 and December 2010. The analyses presented here are based on this subsample of the 3,124 participants. Subjects completed standardized questionnaires collecting information about medical, socio-demographic and life-style factors. Among these 3,124 persons, only 3 lived in nursing homes. Further detailed information about ESTHER has been reported elsewhere [7-9].

Data about OOPP were collected using a short version of a previously used [10,11] health economic questionnaire. In this standardized questionnaire, subjects were asked to report their individual health-related resource utilization during the three-month period preceding the interview in the following sectors: inpatient care, outpatient care, medical supplies, pharmaceuticals, dental prostheses and nursing care. Here, inpatient care included hospital and rehabilitation stays. Outpatient care covered physician and non-physician (e.g. physiotherapy) services and medical supplies are all medical devices that aimed at preventing, alleviating or treating diseases. Pharmaceuticals included both prescription and over-the-counter drugs, and nursing care covered both formal and informal care. Following questions to the respective health care utilization, subjects were then asked to report the individual amount of money they had to pay for the corresponding sector out-of-pocket during the three-month period.

### Out-of-pocket payments

OOPP (synonyms are out-of-pocket expenditures, co-pay-ments) appear in three different forms [3]: As deductibles before the insurance steps in, as additional payments when a stipulated threshold is reached and as direct contributions to costs when utilizing health care services. In the German health care context deductibles chiefly exist in the PHI; additional payments exist mostly for medical supplies and dental prostheses in the SHI, and direct contributions exist only in the SHI (legal co-payment) in all relevant health care sectors. All three types of OOPP were considered in this study.

### Income and burden

Income was measured as disposable, net income from all sources of income after taxes of the entire household. Subjects’ household income was then adjusted in order to account for the household size. For that, the square root equivalence scale was used [12], which divides the total net household income by the square root of household size. Thus, the income of different household sizes became comparable, and synergies of larger households compared to single households were taken into account. As OOPP were collected for a period of three months while the net household income was collected and calculated per month, the latter was multiplied by three in order to keep income and OOPP comparable. The resulting financial burden to patients due to OOPP was measured by the ratio of OOPP and income.

### Illness level

Morbidity was measured by the Cumulative Illness Rating Scale for Geriatrics (CIRS-G) [13], which is a modified version of the Cumulative Illness Rating Scale [14]. The CIRS-G assesses the severity of illnesses in 13 somatic categories and one psychiatric category by 0 to 4 points for each category. One total score is created, consisting of the sum of each category’s points. Likewise, the CIRS-G takes into account both the number of illnesses and their respective severity, combining both to an index that can range (theoretically) from 0 to 56.

### Socio-demographic variables

According to Andersen’s definition of ‘predisposing factors’ for health care utilization [15], the following socio-demographic variables were collected and are considered in this study: age, gender, marital status and education. The self-reported marital status distinguished between single, married, divorced and widowed. The level of education referred to the time of primary and secondary school education and could vary between ‘under ten years’, ‘ten to eleven years’ and ‘more than eleven years’. Additionally, the type of health insurance was considered. Since the number of subjects covered by the above-mentioned governmental schemes is negligible, the status of health insurance could only vary between SHI and PHI, the latter including individuals covered by GS.

### Statistical analyses

All statistical analyses were performed using Stata 11 SE. The level of significance was set at α = 0.05. Differences in means were analyzed using the Student’s t-test. Due to the fact that many respondents reported no OOPP for the preceding three months for sectoral and total OOPP, a two-part model was applied to analyze determinants of OOPP. This is recommended for health expenses data which contain a substantial number of zeros [16]. The first part of the model estimates the probability of having any positive OOPP by means of a logistic regression. In the second part, the expected individual levels of OOPP are calculated only for positive OOPP by means of a general linearized model with gamma distribution and log link function. This model was chosen as the part of the positive values is also positively skewed. Estimators from both models were combined using predictive margins (recycled predictions) [17] in order to regain one total prediction estimator for both models that can be interpreted in terms of OOPP. As the results for widowed individuals were strongly influenced by two outliers with very high OOPP, we deleted them for the two-part regression analyses.

In order to analyze the burden due to OOPP, a generalized linear model for the binomial family was used. A logit link function was chosen that is the canonical link function for generalized linear models for the binomial family. To execute the model, the ‘binreg’ command in Stata was used. The model can predict determinants of proportions and requires a dependent variable ranging from ‘0’ to ‘1’. The share of OOPP on income is a proportion. However, it could occur that total OOPP exceeded the disposable income in the preceding three months. In these cases, when the dependent variable was greater than ‘1’, the values were replaced by ‘1’ for the regression model. Furthermore, income quintiles were constructed and used as control variables in this model. The standard errors were determined by non-parametric bootstrapping (1,000 replications) in order to correct for potentially differently distributed residuals than assumed by the model. The results of this model were reported in terms of odds ratios.

### Missing values

We assumed that participants who left out the question about the amount of money they spent out-of-pocket in the respective health care sector did not spend anything out-of-pocket. As a result, missing values for the variables containing data about OOPP could not occur. For all other variables missing values were imputed using multiple imputation by chained equations in the program ICE (Royston 2004) in Stata 11 SE. A cycle length of 200 was chosen and according to recommendations [18] 100 imputations were executed.

## Results

### Socio-demographic and morbidity data

The sample of the eight-year-follow-up consisted of 3,124 participants, 52.6% being female. The mean age was 69.6 years. The majority of participants (71.8%) was married, 17.4% were widowed, 7.4% divorced and 3.4% single. 66.2% of subjects had passed a school education of less than 10 years, 15.9% 10 or 11 years and 17.9% more than 11 years. Mean net household equivalence income for three months was €4,229. 92.2% of participants were insured by a SHI while 7.8% were insured by a PHI. The average illness level was assessed with a mean CIRS-G of 6.88 points. Table 2 summarizes the socio-demographic characteristics of the sample.

**Table 2:** Sample characteristics

	**All**		**Missings**
Characteristics	n = 3,124		(%)
Gender: n (%)	3,124	(100)	0
- Male	1,481	(47.41)	
- Female	1,643	(52.59)	
Age: mean (SD)	69.63	(6.30)	0
Range	57 - 84		
Marital status: n (%)	3,087	(100)	1.2
- Single	105	(3.40)	
- Married	2,217	(71.82)	
- Divorced	229	(7.42)	
- Widowed	536	(17.36)	
Education: n (%)	3,079	(100)	1.4
- Under 10 years	2,038	(66.19)	
- 10–11 years	550	(17.86)	
- More than 11 years	491	(15.95)	
3-month net income^a^: mean (SD)	4,299.13	(2,053.88)	13.1
Health insurance: n (%)	3,100	(100)	0.8
- Statutory	2,859	(92,23)	
- Private	241	(7.77)	
CIRS-G^b^: mean (SD)	6.88	(5.44)	16.8

^a^Square root equivalence scale; ^b^*CIRS*-*G*: Cumulative Illness Rating Scale for Geriatrics; *SD*: standard deviation.

### Analyses of OOPP

Mean OOPP per capita during three months amounted to €119, with 34% for medical supplies, 22% for dental prostheses, 21% for pharmaceuticals, 17% for outpatient care, 5% for inpatient care and 1% for nursing care. OOPP for medical supplies accounted for one third of total OOPP, and medical supplies plus dental prostheses accounted for more than half of total OOPP. OOPP for nursing care only marginally contributed to total OOPP. Table 3 shows the corresponding values, adding further information about gender-related differences of the considered sample. While in the outpatient sector mean OOPP were significantly higher for women, the differences were not significant for the remaining sectors. Also, the higher total OOPP paid by women (€124) compared to men (€114) did not prove to be statistically significant.

**Table 3:** Mean three-month OOPP per respondent in € (SD), by gender

**Resource**	**All n = 3,124**	**Male n =1,481**	**Female n =1,643**	**p-value**^**a**^
Inpatient care	5.60	5.33	5.84	0.762
	(47.53)	(34.08)	(57.01)	
Outpatient physician and	19.95	17.96	22.54	0.031
Non-physician services	(70.47)	(71.57)	(69.39)	
Medical supplies	40.69	40.42	40.94	0.949
	(224.58)	(239.12)	(210.70)	
Pharmaceuticals	25.61	25.77	25.43	0.858
	(52.92)	(47.13)	(58.69)	
Dental prostheses	26.57	25.06	27.94	0.769
	(274.09)	(312.06)	(234.76)	
Nursing care	1.01	1.01	1.00	0.991
	(20.10)	(19.63)	(20.50)	
Total OOPP	119.14	114.32	124.03	0.477
	(381.10)	(420.53)	(341.75)	

^a^Differences in means: t-test; *SD*: standard deviation.

Table 4 shows the results of the two-part model for OOPP in different sectors as well as for total OOPP. The model was not calculated for OOPP for nursing care since there were only very few positive observations (16 out of 3,124). The model showed a significant positive effect of income on total OOPP, predicting additional OOPP of €11 (p < 0.01) with each €1,000 higher household income. This positive association between income and OOPP could be found in particular for the outpatient sector (p < 0.001) and for pharmaceuticals (p < 0.05). Increasing morbidity – measured by the CIRS-G – was significantly correlated with higher total OOPP with each additional point on the CIRS-G scale being associated with + €3 (p < 0.05). In particular, one additional point on the CIRS-G scale was associated with about €1 (p < 0.05) of additional OOPP for pharmaceuticals. Age significantly influenced OOPP only for pharmaceuticals where every additional year led to about €0.60 higher OOPP (p < 0.05). Widowed individuals paid about €6 (p < 0.05) more than married in the inpatient sector. Furthermore, in the outpatient sector the model predicted significantly lower OOPP for singles (p < 0.05) and men (p < 0.01) as well as for beneficiaries of a PHI (p < 0.05). No significant inequalities in OOPP could be detected for dental prostheses and medical supplies. The coefficients for the combined predictive margins for the educational level did not become significant in either health care sector.

**Table 4:** **Two-part model (1. Logit, 2. GLM**^**a**^**) with OOPP**^**b**^**as dependent variable**

	**Total**			**Inpatient**			**Outpatient**		
**Independent variables**	**Logit OR (SE)**	**GLM b (SE)**	**Predict. margin**	**Logit OR (SE)**	**GLM b (SE)**	**Predict. margin**	**Logit OR (SE)**	**GLM b (SE)**	**Predict. margin**
Male gender	0.92	-0.07	-10.37	1.28	-0.05	1.06	0.89	-0.31**	-7.23**
(*Ref.: Female*)	(0.09)	(0.11)	(13.27)	(0.23)	(0.17)	(1.37)	(0.07)	(0.12)	(2.51)
Private health insurance	0,29***	0.40	4.22	1.05	-0.42	-1,76	0.18	0.56	-7-74*
*(Ref.: Statutory)*	(0.05)	(0.23)	(28.53)	(0.35)	(0.30)	(1.80)	(0.03)	(0.32)	(4.38)
Age	1.00	0.01	1.29	0.99	0.02	0.07	0.98***	-0.01	-0.34
	(0.01)	(0.01)	(1.04)	(0.01)	(0.01)	(0.11)	(0.01)	(0.01)	(0.20)
Education *(Ref.: < 10 y.)*									
- 10–11 years	1.33*	0.09	17.35	0.87*	0.76***	4.23	1.15	0.19	5.16
	(0.17)	(0.14)	(17.87)	(0.21)	(0.23)	(2.64)	(0.12)	(0.15)	(3.38)
- > 11 years	1.18	-0.00	3.92	0.61	0.43	-0.19	1.15	0.30	7.98
	(0.17)	(0.16)	(18.79)	(0.18)	(0.28)	(1.80)	(0.14)	(0.17)	(4.21)
Illness level – CIRS-G^c^	1.00	0.03*	3.12*	1.03*	0.01	0.26	0.99	0.01	0.03
	(0.01)	(0.01)	(1.27)	(0.02)	(0.02)	(0.13)	(0.01)	(0.01)	(0.23)
Marital status *(Ref: married)*									
- Single	0.81	0.40	46.24	2.06	-0.18	2.82	0.94	-0.70*	-10.75*
	(0.19)	(0.31)	(45.87)	(0.76)	(0.35)	(3.34)	(0.19)	(0.31)	(3.43)
- Divorced	0.87	0.08	5.72	0.98	0.06	0.19	0.86	-0.16	-4.31
	(0.15)	(0.21)	(23.56)	(0.36)	(0.33)	(2.18)	(0.13)	(0.22)	(3.86)
- Widowed	1.08	0.25	33.62	1.54	0.48*	6.24*	1.01	0.06	1.48
	(0.15)	(0.15)	(20.78)	(0.36)	(0.22)	(2.87)	(0.11)	(0.16)	(3.66)
Income^d^	1.03	0.09**	11.15**	1.09	-0.04	0.25	1.07**	0.12***	3.11***
	(0.03)	(0.03)	(3.80)	(0.05)	(0.05)	(0.36)	(0.02)	(0.03)	(0.73)
Constant		3.55***			2.84**			3.47***	
		(0.62)			(0.95)			(0.68)	
N	3,122	3,122	3,122	3,122	3,122	3,122	3,122	3,122	3,122
		**Medical supplies**			**Dental prostheses**			**Pharmaceuticals**	
**Independent variables**	**Logit OR (SE)**	**GLM b (SE)**	**Predict. margin**	**Logit OR (SE)**	**GLM b (SE)**	**Predict. margin**	**Logit OR (SE)**	**GLM b (SE)**	**Predict. margin**
Male gender	0.87	0.22	4.14	0.93	-0.28	-9.06	0.99	-0.09	-2.84
*(Ref.: Female)*	(0.09)	(0.21)	(9.08)	(0.19)	(0.26)	(8.65)	(0.08)	(0.07)	(2.41)
Private health insurance	0.63*	0.11	-9.62	0.29*	2.12**	35.64	0.52***	0.62***	11.84
*(Ref.: Statutory)*	(0.14)	(0.42)	(14.51)	(0.18)	(0.79)	(58.06)	(0.08)	(0.15)	(8.96)
Age	1.01	-0.00	0.19	1.03	-0.01	0.32	1.00	0.02***	0.59*
	(0.01)	(0.02)	(0.69)	(0.02)	(0.02)	(0.65)	(0.01)	(0.01)	(0.19)
Education *(Ref.: < 10 y.)*									
- 10–11 years	1.01	-0.13	-4.84	1.17	0.16	9.85	1.08	-0.03	0.64
	(0.13)	(0.25)	(10.20)	(0.28)	(0.32)	(13.47)	(0.11)	(0.09)	(3.20)
- > 11 years	0.87	-0.06	-6.79	0.78	-0.21	-9.64	1.07	0.04	0.83
	(0.13)	(0.30)	(11.78)	(0.25)	(0.40)	(10.31)	(0.13)	(0.10)	(3.47)
Illness level – CIRS-G^c^	1.01	-0.02	-0.43	1.00	0.05*	1.33	1.02	0.03***	0.98*
	(0.01)	(0.02)	(0.83)	(0.02)	(0.02)	(0.83)	(0.01)	(0.01)	(0.24)
Marital status *(Ref: married)*									
- Single	0.97	0.37	18.52	1.08	1.04	46.84	0.78	0.13	4.07
	(0.27)	(0.66)	(30.21)	(0.57)	(0.67)	(57.3)	(0.16)	(0.19)	(9.05)
- Divorced	1.15	-0.08	2.27	1.43	-0.11	5.94	0.88	0.14	2.68
	(0.21)	(0.42)	(13.87)	(0.49)	(0.46)	(15.72)	(0.13)	(0.13)	(4.71)
- Widowed	1.49**	0.34	32.23	1.25	0.06	7.10	1.16	-0.03	1.11
	(0.19)	(0.30)	(17.02)	(0.31)	(0.32)	(11.36)	(0.13)	(0.09)	(3.21)
Income^d^	1.02	0.07	3.69	1.06	0.08	3.60	1.02	0.05**	1.74*
	(0.03)	(0.06)	(2.43)	(0.06)	(0.08)	(2.66)	(0.02)	(0.02)	(0.69)
Constant		5.17***			6.48***			1.51***	
		(1.21)			(1.31)			(0.39)	
N	3,122	3,122	3,122	3,122	3,122	3,122	3,122	3,122	3,122

^a^Generalized linear model with log link and gamma distribution ^b^out-of-pocket payments ^c^Cumulative Illness Rating Scale for Geriatrics ^d^3-month net income in €1,000 (square root equivalence scale); OR: Odds ratio; SE: Standard error *p < .05 **p < .01 ***p < .001.

### Analyses of financial burden due to OOPP

The average burden of OOPP, measured as the ratio of OOPP and income (square root equivalence scale), was 3.23% (standard deviation: 10.99%). The burden ranged from 0% for patients without OOPP in the last three months to 310%, indicating that OOPP exceeded the household income by more than three times for the maximally burdened. The very high financial burden probably resulted from the short observation period (three months). Thus, the purchase of an expensive medical product falling in this period of time, e.g. new glasses or dental prostheses, could have led to the observed values.

The generalized linear model for the binomial family with logit link predicted a higher burden for women and SHI-beneficiaries, yet without statistically significant effects (Table 5). Likewise, neither the illness level nor the educational level influenced the burden significantly. However, the model showed a significantly higher burden for widowed as compared to married individuals. The burden of widowed individuals exceeded those of married persons by about 42% (p < 0.05). This significant association also persisted after excluding the two widowed outliers with very high OOPP from regression analysis, although then widowed individuals were predicted to be burdened more than married individuals by only about 32%. Compared to the lowest income quintile, all higher income quintiles showed a lower burden. The wealthiest income quintile was predicted to be burdened by OOPP of about 52% less than the poorest income quintile (p < 0.01). Additionally, age was positively associated with a higher financial burden, with each additional year being associated with a 1.7% higher burden (p < 0.05).

**Table 5:** **Generalized linear model for the binomial family (logit link) with financial burden**^**a**^**as dependent variable**

**Independent predictor variables**	**Odds ratio**	**95% CI**	
Male gender (*reference: female*)	0.880	(0.708	1.093)
Private health insurance *(reference: SHI)*	0.890	(0.564	1.405)
Age	1.017*	(1.001	1.034)
Education 10–11 years (*reference: <10 years)*	1.090	(0.842	1.410)
>11 years	0.929	(0.693	1.245)
Illness level – CIRS-G^b^	1.020	(0.998	1.042)
Marital status: Single *(reference: married)*	1.619	(0.812	3.231)
Divorced	1.081	(0.746	1.565)
Widowed	1.418*	(1.070	1.880)
Income quintile 2 (*reference: quintile 1*)	0.708	(0.478	1.047)
Income quintile 3	0.737	(0.520	1.044)
Income quintile 4	0.715	(0.503	1.016)
Income quintile 5	0.478**	(0.311	0.733)
N	3,124		

^a^Burden: OOPP to income ratio; ^b^*CIRS-G*: Cumulative Illness Rating Scale for Geriatrics; *p < .05 **p < .01; *CI*: bias-corrected bootstrapped confidence intervals (1,000 replications).

## Discussion

### Main findings

The aim of this study was to analyze inequalities in OOPP among elderly Germans. Main findings were, firstly, that the greatest share of mean OOPP of €119 for a three-month period were paid for medical supplies, dental prostheses and pharmaceuticals. Total OOPP amounted to 3.23% of disposable income (square root equivalence scale). Secondly, higher income was associated with higher total OOPP, amounting to + €11 of total OOPP per each €1,000 higher income. Nonetheless, the wealthiest quintile had a significantly smaller financial burden than the poorest one. Thirdly, an increasing illness level was associated with higher total OOPP. And fourthly, age was associated with a higher burden and significant sectoral lower OOPP could be detected for men and single individuals as compared to married persons in the outpatient sector as well as for PHI compared to SHI beneficiaries.

### Hypotheses on causes

#### Extent and structure of OOPP

Most OOPP were paid for medical supplies. OOPP for SHI-beneficiaries for medical supplies could occur in three different manners. Firstly, there was the legal co-payment that did not exceed €10 per month or prescription respectively (see Table 1). Secondly, costs for glasses had to be borne totally by beneficiaries. Thirdly, medical supplies that went beyond pure medical necessity (e.g. electrical wheelchairs) were only reimbursed to the extent of the costs of a conventional product, resulting in OOPP accounting for the residual amount. It appears probable that the OOPP for glasses and medical supplies that go beyond the legally stipulated amount for common products mainly caused the relatively high OOPP in this sector. Glasses are almost always used in the elderly and – even if normally seldom bought – can be rather costly. Also, medical supplies that went beyond pure medical necessity could reach very high amounts (e.g. in case an electrical wheelchair is purchased), leading – even if only rarely occurring for respondents in the three months – to high mean OOPP (and a highly skewed distribution of OOPP for medical supplies).

OOPP for dental prostheses were second highest. In general, dental prostheses were only reimbursed for SHI-beneficiaries to the half of costs. In contrast to all the other remaining sectors, OOPP were not limited to 10% of costs or €10 per unit, leading to relatively high OOPP in this sector. The third highest amount of OOPP was paid for pharmaceuticals, for which legal co-payments were limited to €10 per pack of medication. Additionally to a legal co-payment, OOPP for non-prescription drugs, which are not SHI-reimbursed at all, might explain the size of OOPP for pharmaceuticals.

The structure of sectoral components of total OOPP suggests that most OOPP were paid in sectors in which the SHI has completely or partially withdrawn from reimbursing health goods and services (i.e. glasses, dental prostheses, non-prescription drugs). The SHI legal co-payments did not appear to be the main cost-driver for total OOPP in the elderly cohort. As a result, there was a discrepancy between the structure of officially reported legal SHI-co-payments [19] and the structure of all OOPP in our cohort. Officially reported legal SHI-co-payments were led by outpatient care followed by pharmaceuticals and the inpatient sector in 2009.

### Income

Increasing income was correlated with higher OOPP (Table 4). Potentially, the influence of the above described 2%- or 1%-income-threshold could partially contribute to an explanation of the described phenomenon by limiting OOPP for goods and services reimbursed by the SHI for individuals with low income. Nevertheless, the 2%- respectively 1%-threshold for legal co-payments did not show a preventing effect on the burden of different income quintiles as the lowest quintile had a higher burden than the highest.

Higher income led to higher OOPP especially in the outpatient sector, where wealthier people could potentially utilize certain so-called non-covered individual health services (IHS) more often. These are services that go beyond legally stipulated patient care and must be completely paid by the patient. Examples for IHS are the measurement of the eye pressure without certain medical indication by an eye specialist or professional tooth cleaning by a dentist. As it is the nature of these services that they are not completely necessary from a medical point of view, it appears probable that wealthier people afforded more of them whereas poorer patients avoided them, leading to the observed positive association of income and OOPP.

### Illness level

The illness level – measured by the CIRS-G – had a significant effect on total OOPP. This appears to be natural as a higher illness level leads to a higher need and thus a higher demand for health care services. And – following this crude logic – higher demand leads to higher resource utilization that entails higher corresponding OOPP. This association between illness level and OOPP could in particular been seen for pharmaceuticals.

However, for all other sectors this does not apply. This finding might be explained by the high variance of OOPP in these sectors (e.g. medicals supplies and dental prostheses). Likewise, days in hospitals are rare events, leading to the same effect of a very high variance for the regarded three-month period, eventually preventing the positive association between CIRS-G and inpatient OOPP from being statistically significant.

### Other socio-demographic variables

Age was associated significantly with a higher financial burden. This might be the result of decreasing income with higher age. Besides, higher age normally is associated with a lower health status that is not completely captured by the CIRS-G, leading to higher service use and corresponding OOPP.

Women and single persons paid significantly more OOPP in the outpatient sector than men and married individuals respectively (see Table 4). There are two potential causes for that. Firstly, a different structure of resource utilization by certain subgroups compared to others could entail a different corresponding size of OOPP. For example, it is well known for the German health care context that women utilize outpatient physician services more frequently than men do [20]. Higher service utilization could thus lead in this sector to higher OOPP for women. Yet, in this example, a SHI-insured woman only had to pay €10 per quarter for the first outpatient physician contact regardless of the concrete amount of contacts in one quarter (Table 1). And as virtually all participants had at least one contact with a physician gender-related differences can hardly result from this legal co-payment of €10 per quarter. However, patients could moreover utilize the above-described IHS, which could explain the gender-related inequality for outpatient care. Additionally, the regulation scheme for OOPP in the outpatient non-physician part – for which patients had to pay €10 per prescription and 10% of costs – could also contribute to an explanation of the observed inequalities. Due to the payable OOPP per contact for non-physicians higher resource utilization by women could also have contributed to the resulting gender-related inequalities.

These explanations illustrate that deviating resource utilization of certain subgroups and corresponding legal regulation of OOPP must be considered simultaneously in order to explain detected inequalities. Likewise, the fact that PHI beneficiaries paid lower OOPP in the outpatient sector would be explained by the existence of the quarterly co-payment of €10 for SHI beneficiaries that PHI beneficiaries did not have to pay. Moreover, PHI beneficiaries did not have to pay OOPP for non-physician services so that they were burdened much less in this sector.

### International comparison

Due to a long tradition with a comprehensive system of OOPP, most published studies focus on inequalities in OOPP in the United States (US) [3], often with special regard towards OOPP for pharmaceuticals [21-27]. Thus, in the following the main findings of this study were compared to evidence from the US.

In contrast to the US, OOPP on pharmaceuticals did not appear to be crucial for inequalities in OOPP and the burden in Germany. Thus, in our study, mean OOPP for pharmaceuticals only accounted for 21% of average OOPP. Besides, only few determinants, such as morbidity, age and income significantly influenced OOPP for pharmaceuticals, whilst in the US more determinants like gender could be identified [25]. The importance of the pharmaceutical sector for inequalities in OOPP in the US is in contrast to the findings in Germany and most likely resulted from a lower, varying insurance coverage of pharmaceuticals in the US. In Germany, irrespective of type of health insurance, all beneficiaries profited from a very comprehensive coverage of pharmaceutical expenses. Likewise, the often investigated problem of non-adherence due to financial reason [21,27] appears to be rather implausible for the German health care context.

The average financial burden due to OOPP in the elderly appeared to be much lower in Germany than in the US. Whilst in this population-based cohort study it was about three percent, Crystal et al. reported a mean burden for the large group of Medicare beneficiaries of 19 percent [28]. In our study, the type of health insurance did neither influence total OOPP nor the resulting burden significantly. This is in contrast to findings in the US where the type of insurance decisively influenced the financial burden [29-31]. This probably is a result of a much larger heterogeneity among the different types of health insurances available for the elderly in the US, whereas both PHI and SHI in Germany are obliged to offer a comprehensive coverage, especially for the in- and outpatient sector and pharmaceuticals.

### Strengths and limitations

The analyses are based on a large cohort of elderly Germans. At baseline recruitment from 2000 to 2002 participants were almost representative with regard to age and gender for the entire population aged 50 to 74 in the Saarland, Germany [8]. From ESTHER-baseline to the eight-year follow-up 499 participants deceased and 505 people were no longer able to participate for health reasons. This might have led to an underestimation of the illness level in the ESTHER-sample as compared to the entire elderly population. Besides, only 3 individuals living in nursing homes could be recruited for the sample, leading to low mean OOPP for nursing care in our study.

Also limiting is the high skewedness of distribution of OOPP. Arguably, the short period of time participants had to report OOPP might have contributed to the highly positively skewed distribution of OOPP. Rare events like the purchase of (expensive) medical supplies might have fallen in this recall period only for very few subjects whilst a vast majority had little or no OOPP. Due to this relatively small recall period in the questionnaire, the variance of OOPP became high, probably preventing existing correlations from being observed by the used models. On the other hand, the small recall period is likely to have minimized a potential recall bias.

## Conclusion

Despite an – in comparison to the US – relatively small burden of OOPP in the elderly German population today, decision makers will have to continue focusing on resulting inequalities due to OOPP. Medical progress as well as the demographic change in future Germany will certainly lead to rising OOPP in the long term. The present study can help decision makers by stating current inequalities, discussing the mechanism causing them and thus making decision makers to take them into account when adapting OOPP-relevant regulations.

## Abbreviations

OECD: Organization for economic co-operation and development; OOPP: Out-of-pocket payments; SHI: Statutory health insurance; PHI: Private health insurance; GS: Governmental schemes; ESTHER: Epidemiologische Studie zu Chancen der Verhütung, Früherkennung und optimierten Therapie chronischer Erkrankungen in der älteren Bevölkerung; CIRS-G: Cumulative illness rating scale for geriatrics; SD: Standard deviation; GLM: Generalized linear model; OR: Odds ratio; SE: Standard error; GLM: Generalized linear model; CI: Confidence interval; IHS: Individual health services; US: United States.

## Competing interests

All authors declare that they have no competing interests.

## Authors’ contributions

JOB performed the statistical analyses and drafted the manuscript. HM participated in the design of the study. HB, BW, WEH, RQ contributed to data acquisition and revised the manuscript critically. KUS revised the manuscript critically and participated in the coordination of this study. DH conceived the study, participated in the design of the study, in performing the statistical analysis and in drafting the manuscript. HHK conceived the study and participated in its design and coordination and helped to draft the manuscript. All authors read and approved the final manuscript.

## References

[B1] OECD Health Data 2012 2012 Paris: Organization for Economic Co-operation and Development 2012

[B2] Tambor M Pavlova M Woch P Groot W Diversity and dynamics of patient cost-sharing for physicians’ and social services in the 27 European Union countries Eur J Public Health 2011 21 5 585 590 10.1093/eurpub/ckq139 20884659

[B3] Corrieri S Heider D Matschinger H Lehnert T Raum E König HH Income-, education- and gender-related inequalities in out-of-pocket health-care payments for 65+ patients - a systematic review Int J Equity Health 2010 9 20 1 11 20701794 10.1186/1475-9276-9-20 PMC2925341

[B4] OECD OECD Factbook 2011–2012 2012 Paris: Economic, Environmental and Social Statistics

[B5] Statistisches Bundesamt Bevölkerung Deutschlands bis 2060–12. koordinierte Bevölkerungsvorausberechnung 2009 Wiesbaden: Statistisches Bundesamt

[B6] Informationsblatt zu den Zuzahlungsregelungen der Gesetzlichen Krankenversicherung http://www.bmg.bund.de/krankenversicherung/arzneimittelversorgung/zuzahlung.html

[B7] Raum E Rothenbacher D Löw M Stegmaier C Ziegler H Brenner H Changes of cardiovascular risk factors and their implications in subsequent birth cohorts of older adults in Germany: a life course approach Eur J Cardiovasc Prev Rehabil 2007 14 6 809 814 10.1097/HJR.0b013e3282eeb308 18043304

[B8] Löw M Stegmaier C Ziegler H Rothenbacher D Brenner H Epidemiological investigations of the chances of preventing, recognizing early and optimally treating chronic diseases in an elderly population (ESTHER study) Dtsch Med Wochenschr 2004 129 49 2643 2647 10.1055/s-2004-836089 15578318

[B9] Schöttker B Haug U Schomburg L Köhrle J Perna L Müller H Holleczek B Brenner H Strong associations of 25-hydroxyvitamin D concentrations with all-cause, cardiovascular, cancer, and respiratory disease mortality in a large cohort study Am J Clin Nutr 2013 97 4 782 793 10.3945/ajcn.112.047712 23446902

[B10] König HH Born A Heider D Matschinger H Heinrich S Riedel-Heller SG Surall D Angermeyer MC Roick C Cost-effectiveness of a primary care model for anxiety disorders Br J Psychiatry 2009 195 4 308 317 10.1192/bjp.bp.108.058032 19794198

[B11] Roick C Kilian R Matschinger H Bernert S Mory C Angermeyer MC German adaptation of the client sociodemographic and service receipt inventory - an instrument for the cost of mental health care Psychiatr Prax 2001 28 Suppl 2 S84 S90 11605129 10.1055/s-2001-17790

[B12] What are equivalence scales? http://www.oecd.org/eco/growth/OECD-Note-EquivalenceScales.pdf

[B13] Miller M Paradis C Houck P Mazumdar S Stack J Rifai A Mulsant B Reynolds C Rating chronic medical illness burden in geropsychiatric practice and research: application of the Cumulative Illness Rating Scale Psychiatry Res 1992 41 3 237 248 10.1016/0165-1781(92)90005-N 1594710

[B14] Linn BS Linn MW Gurel L Cumulative illness rating scale J Am Geriatr Soc 1968 16 5 622 626 5646906 10.1111/j.1532-5415.1968.tb02103.x

[B15] Andersen R Newman JF Societal and individual determinants of medical care utilization in the United States Milbank Mem Fund Q Health Soc 1973 51 1 95 124 10.2307/3349613 4198894

[B16] Manning WG Basu A Mullahy J Generalized modeling approaches to risk adjustment of skewed outcomes data J Health Econ 2005 24 3 465 488 10.1016/j.jhealeco.2004.09.011 15811539

[B17] Lê Cook B McGuire TG Lock K Zaslavsky AM Comparing methods of racial and ethnic disparities measurement across different settings of mental health care Health Serv Res 2010 45 3 825 847 10.1111/j.1475-6773.2010.01100.x 20337739 PMC2875762

[B18] Graham JW Olchowski AE Gilreath TD How many imputations are really needed? Some practical clarifications of multiple imputation theory Prev Sci 2007 8 3 206 213 10.1007/s11121-007-0070-9 17549635

[B19] Gesetzliche Krankenversicherung - Vorläufige Rechnungsergebnisse 4. Quartal 2009 [http://www.bmg.bund.de/fileadmin/redaktion/pdf_statistiken/krankenversicherung/KV-45-4-Quartal-2009.pdf]

[B20] Thode N Bergmann E Kamtsiuris P Kurth BM Predictors for ambulatory medical care utilization in Germany Bundesgesundheitsblatt Gesundheitsforschung Gesundheitsschutz 2005 48 3 296 306 10.1007/s00103-004-1004-3 15768302

[B21] Soumerai SB Pierre-Jacques M Zhang F Ross-Degnan D Adams AS Gurwitz J Adler G Safran DG Cost-related medication nonadherence among elderly and disabled medicare beneficiaries: a national survey 1 year before the medicare drug benefit Arch Intern Med 2006 166 17 1829 1835 10.1001/archinte.166.17.1829 17000938

[B22] Sambamoorthi U Shea D Crystal S Total and out-of-pocket expenditures for prescription drugs among older persons Gerontologist 2003 43 3 345 359 10.1093/geront/43.3.345 12810898

[B23] Klein D Turvey C Wallace R Elders who delay medication because of cost: health insurance, demographic, health, and financial correlates Gerontologist 2004 44 6 779 787 10.1093/geront/44.6.779 15611214

[B24] Rogowski J Lillard LA Kington R The financial burden of prescription drug use among elderly persons Gerontologist 1997 37 4 475 482 10.1093/geront/37.4.475 9279036

[B25] Fahlman C Lynn J Doberman D Gabel J Finch M Prescription drug spending for Medicare + Choice beneficiaries in the last year of life J Palliat Med 2006 9 4 884 893 10.1089/jpm.2006.9.884 16910803

[B26] Gellad WF Huskamp HA Phillips KA Haas JS How the new medicare drug benefit could affect vulnerable populations Health Aff (Millwood) 2006 25 1 248 255 10.1377/hlthaff.25.1.248 16403761 PMC1403812

[B27] Rector TS Venus PJ Do drug benefits help Medicare beneficiaries afford prescribed drugs? Health Aff (Millwood) 2004 23 4 213 222 10.1377/hlthaff.23.4.213 15318583

[B28] Crystal S Johnson RW Harman J Sambamoorthi U Kumar R Out-of-pocket health care costs among older Americans J Gerontol B Psychol Sci Soc Sci 2000 55 1 S51 S62 10728130 10.1093/geronb/55.1.s51

[B29] Adams AS Soumerai SB Ross-Degnan D Use of antihypertensive drugs by Medicare enrollees: does type of drug coverage matter? Health Aff (Millwood) 2001 20 1 276 286 10.1377/hlthaff.20.1.276 11194852

[B30] Banthin JS Cunningham P Bernard DM Financial burden of health care, 2001–2004 Health Aff (Millwood) 2008 27 1 188 195 10.1377/hlthaff.27.1.188 18180494

[B31] Pourat N Rice T Kominski G Snyder RE Socioeconomic differences in Medicare supplemental coverage Health Aff (Millwood) 2000 19 5 186 196 10.1377/hlthaff.19.5.186 10992668

